# Nutritional Aspects of Juvenile Idiopathic Arthritis: An A to Z for Dietitians

**DOI:** 10.3390/children10020203

**Published:** 2023-01-23

**Authors:** Maria G. Grammatikopoulou, Konstantinos Gkiouras, Vasiliki Syrmou, Tonia Vassilakou, Theodora Simopoulou, Chistina G. Katsiari, Dimitrios G. Goulis, Dimitrios P. Bogdanos

**Affiliations:** 1 Immunonutrition Unit, Department of Rheumatology and Clinical Immunology, University General Hospital of Larissa, Faculty of Medicine, School of Health Sciences, University of Thessaly, Biopolis, GR-41110 Larissa, Greece; 2 Department of Public Health Policy, School of Public Health, University of West Attica, 196 Alexandras Avenue, GR-11521 Athens, Greece; 3 Unit of Reproductive Endocrinology, 1st Department of Obstetrics and Gynecology, Medical School, Faculty of Health Sciences, Papageorgiou General Hospital, Aristotle University of Thessaloniki, 76 Agiou Pavlou Str., Pavlos Melas, GR-56429 Thessaloniki, Greece

**Keywords:** bone mineral density, rheumatoid arthritis, resting energy expenditure, short stature, malnutrition, immunonutrition, body composition, JADAS-27

## Abstract

Juvenile idiopathic arthritis (JIA) represents a chronic, autoimmune, rheumatic musculoskeletal disease with a diagnosis before 16 years of age. Chronic arthritis is a common manifestation in all JIA subtypes. The nature of JIA, in combination to its therapy often results in the development of nutrition-, gastrointestinal (GI)- or metabolic-related issues. The most-common therapy-related nutritional issues involve methotrexate (MTX) and glucocorticosteroids (GCC) adverse events. MTX is a folic acid antagonist, thus supplementation with folic acid in required for improving GI side effects and correcting low serum levels. On the other hand, long-term GCC administration is often associated with hyperglycemia, insulin resistance and growth delay. This relationship is further aggravated when more joints are affected and greater doses of GCC are being administered. Apart from stature, body mass index z-scores are also suboptimal in JIA. Other signs of malnutrition include decreased phase angle and muscle mass, especially among patients with polyarthritis JIA. Evidence also points to the existence of an inverse relationship between disease activity and overweight/obesity. Specific dietary patterns, including the anti-inflammatory diet, might confer improvements in selected JIA outcomes, but the level of available research is yet insufficient to draw safe conclusions. The majority of patients exhibit suboptimal vitamin D status; hence, supplementation is recommended. Collectively, the evidence indicates that, due to the age of onset and the complexity of the disease, along with its pharmacotherapy, children with JIA are prone to the development of several nutritional problems, warranting expert monitoring. Vitamin deficiencies, oral and GI-problems limiting dietary intake, faltering growth, overweight and obesity, physical inactivity, or impaired bone health are among the many nutritional issues in JIA requiring dietitian support.

## 1. Introduction

Juvenile idiopathic arthritis (JIA) is an umbrella term, encompassing heterogenous arthritides with an onset before 16 years of age [1]. It consists of the most common chronic, autoimmune, rheumatic and musculoskeletal disease (RMD) diagnosis of unknown etiology encountered during childhood [2]. Recent epidemiological data indicate that approximately two million children are affected by JIA globally [3], with the condition being more prevalent among girls than boys [4].

Based on the most recent classification seven distinct JIA subtypes exist [1], namely (a) systemic-onset JIA (sJIA), characterized by systemic features including a spiking fever and arthritis, (b) polyarthritis, affecting ≥ 5 joints, which can be rheumatoid-factor (RF)-negative, or (c) RF-positive, (d) oligoarthritis, featuring arthritis affecting < 5 joints during the first six months of the disease onset, (e) enthesitis-related arthritis (ERA), (f) psoriatic arthritis, and (f) undifferentiated arthritis. Patients with different subtypes exhibit characteristic extra-articular and systemic manifestations, but chronic arthritis is the common manifestation among all subtypes [5].

The nature of JIA, the young age of onset and the multiple medications required for its management have multiple effects on the growth and the nutritional status of patients. In this manner, understanding the nutritional issues associated with the diagnosis is important for their early identification and their management, in order to improve the health and prognosis of affected children.

## 2. JIA Treatment

The medical management of JIA aims in tampering down disease activity, normalizing joint function and preventing joint damage, while attaining an optimum growth velocity [6].

During the past decades, JIA management was heavily dependent on the use of corticosteroids (CCS) and few disease-modifying anti-rheumatic drugs (DMARDs), such as methotrexate (MTX) or sulfasalazine (SSZ) [7]. Their use induced a variety of dose-dependent side effects, leading to the inadequate control of inflammation in some cases, joint damage and growth retardation [8]. The new class of synthetic, targeted therapeutic agents, called biologic agents, transformed the landscape in JIA management [8]. Combination therapy of MTX with biologics is also recommended, for improved treatment efficacy [9].

### 2.1. Medication-Induced Nutrition-Related Side Effects

#### 2.1.1. MTX Adverse Events

Although MTX is the drug of choice in JIA, despite its importance, it is associated with increased gastrointestinal (GI) intolerance [10,11] and vitamin deficiencies. In fact, when patients decide to discontinue MTX, the main reported reason is the induced GI toxicity [10]. GI reflux, diarrhea, abdominal pain, constipation and indigestion [12] consist of common MTX adverse events, with increasing severity in higher treatment doses.

MTX is a folic acid antagonist, and as a result, MTX treatment is associated with suboptimal folic acid concentrations [13,14]. With this in mind, oral nutrient supplementation (ONS) with folate is subscribed in parallel to MTX [15]. Meta-analyses reveal that folic acid supplementation can reduce hepatotoxicity and the frequency of GI side-effects associated with MTX, lower transaminases levels and limit the number of patients choosing to discontinue MTX [16]. In parallel, the low folic acid levels associated with MTX treatment are also responsible for the elevated circulating concentrations of homocysteine (HCY) observed among patients with RMDs (including JIA) on MTX, compared to those on anti-tumor necrosis factor-α (anti-TNF-α) treatment [16,17,18,19,20]. HCY reduces the activity of the asymmetric dimethylarginine (ADMA) decomposing enzyme and carotid intima-media thickness, initiating atheromatosis and atherosclerosis, while multiplying the cardiovascular disease (CVD) risk of patients, irrespective of the methylenetetrahydrofolate reductase (*MTHFR*) C677T genotype [17,21]. Subsequently, folic acid ONS can effectively lower serum HCY concentrations and lessen the future incidence of stroke [22].

Given the adverse effects of MTX, the parent and patient voting panel participating in the American College of Rheumatology/Arthritis Foundation clinical practice guidelines reported that they wished to be made aware of available MTX alternatives [23]. Furthermore, the Methotrexate Advice and Recommended Actions on Juvenile Idiopathic Arthritis (MARAJIA) expert group recommends ONS with folic/folinic acid for the prevention of MTX side effects [24]. The advised dose corresponds to 1/3 of the respective MTX dose for folinic acid, administered >24 h after the weekly MTX dose [24]. For folic acid, the intake of 1 mg/day is advised, skipping the day when MTX is being administered [24].

#### 2.1.2. Recombinant Human Growth Hormone (rhGH) Side-Effects

The inadequate growth velocity and short stature frequently observed in children with JIA is routinely treated with recombinant human growth hormone (rhGH) [25,26]. Treatment with rhGH is associated with a plethora of side-effects, including JIA relapse [27], as the direct result of rhGH stimulation of the macrophage cytokines release which, in turn, is involved in the production of interferon-c (IFN-c) by the T lymphocytes [28].

Furthermore, rhGH therapy reduces glucose tolerance and increases the concentration of glycosylated hemoglobin (HbA1c), as a result of the induced hyperinsulinemia [29]. The actions of rhGH affect multiple tissues including the muscles, liver, fat and pancreas, either in a direct or indirect manner, via complex interactions with insulin and insulin growth factor -1 (IGF-1), all propelling a diabetogenic action [30,31]. According to a recent systematic review [25], “glucose intolerance” and diabetes are among the most common adverse events of rhGH administration in children with JIA.

#### 2.1.3. Glucocorticosteroid (GCC)-Related Adverse Events

In rheumatology, high glucocorticosteroid (GCC) doses are often prescribed and their long-term use has been associated with increased bone resorption and risk for osteoporosis [32,33]. Furthermore, GCCs are also interfering with growth through the growth hormone (GH)/IGF-1 axis [34] and, as a result, long-term GCC administration may induce negative effects on the stature of children with a JIA diagnosis [35,36,37].

In parallel, GCCs antagonize the insulin response by promoting liver gluconeogenesis and inhibiting peripheral glucose uptake, in both the skeletal muscle and the white adipose tissue [38]. As a result, prolonged, high doses of GCCs promote hyperglycemia and insulin resistance (IR). In turn, hyperglycemia may lower the efficacy of JIA therapy through the induction and sustenance of inflammation [39]. Children with sJIA, in particular those who are overweight and/or obese, demonstrate increased IR and subsequently, a greater risk for developing diabetes mellitus (DM) in the future [40].

## 3. Malnutrition

Malnutrition is an umbrella term spanning from undernutrition (including underweight and short stature), to overnutrition (overweight and obesity). Children with JIA are particularly prone to all forms of malnutrition, due to the synergistic effects of disease manifestations, low physical activity (PA) levels and medication-induced adverse events. 

### 3.1. Growth Delay in JIA

The World Health Organization (WHO) [41] defines short stature as a severe form of undernutrition, diagnosed in children with a height-for-age z-score (HAZ) < -2 standard deviations (SD). In JIA, short stature [42], inability to reach height according to the genetic (parental) potential, growth retardation and delayed puberty are common findings [43] for reasons beyond the nutritional intake of patients. The observed growth delay is, in fact, the residue of reduced growth of the lower extremities in particular, with rare cases of retarded growth of the spinal column [44]. Subsequently, many children with JIA have infantile body proportions [44,45]. Growth retardation is further augmented in sJIA, and in children with a greater number of affected joints [45,46]. The etiology of growth retardation is multifactorial, stemming from the frequent infections, the stress associated with chronic illness, the inflammation, the underlying malnutrition and altered body composition, the adverse events of therapy (including CCS treatment), and the delay in pubertal onset, or the slow pubertal progression [44,47,48]. These factors act additively in deteriorating linear growth, by exerting a systemic effect on the GH/IGF-1 and the gonadotropin-releasing hormone (GnRH)-gonadotropin-gonadic axis, and through acting on the growth plate homeostasis and function [29,47]. As a result, it is often difficult to separate the degree of growth retardation attributed to the disease itself and that associated with the adverse events of the treatment [44,49]. Studies assessing growth in children with JIA are presented in Table 1.

According to Saha et al. [46], a year preceding the JIA diagnosis, children are generally taller than their healthy peers, growing at a faster rate. Post-diagnosis, a decline in growth is noted during the first year, with children with polyarticular disease being the most affected [46]. Similar results were also reported by the Childhood Arthritis Prospective Study (CAPS) cohort [55], where children with JIA were followed for three years after their initial presentation to the rheumatology clinic. Although height at three years was within the population norm for most patients, as a cohort, youngsters with JIA demonstrated a growth reduction in height over the first three years of disease diagnosis [55]. Interestingly, patients with the lowest HAZ at presentation were the most likely to show improvement at three years [55].

Miyamae [35] suggested that in sJIA HAZ is inversely correlated with the duration of the disease. Apart from disease duration however, the age of puberty, and the higher degree of functional joint involvement have been shown to act synergistically in augmenting growth impairment in CCS-naive patients with systemic and polyarticular JIA [56].

Nonetheless, research is unanimous on the deleterious effects of CCS use on the stature of children with JIA [35,36,37]. In an early study [57], a significant loss of height was observed (exceeding two standard deviations [SD) during the first years post-JIA diagnosis. This height loss correlated well with the duration of prednisone (PRED) treatment. After remission was induced and PRED therapy was discontinued, the majority (70%) of patients achieved catch-up growth but some (30%) retained a persistent loss of stature. The mean final height of participants was correlated with height at the end of steroid treatment and was different between the children who achieved catch-up growth and those who did not [57]. In parallel, at prepubertal stages [37] HAZ has been shown to differ between patients and controls. Polyarticular JIA and greater cumulative GCC doses have been shown to synergistically contribute to short stature [37]. It appears that, long post-GCC cessation, children with polyarticular/sJIA still remain susceptible to low height and delayed puberty [37]. Apart from disrupting growth through the GH/IGF1 axis [34], prolonged GCC treatment also alters the secretion and pulsatility of GH, through the somatostatin inhibitor tone and by reducing the expression of GH receptors through the Janus kinase (JAK)-2/signal transducers and activators of the transcription (STAT)-5 pathway, tamping down IGF-1 mRNA levels within the liver cells [34,62]. In a comparative study of patients on different CCS treatment durations, Wang et al. [61] showed that continuous systemic CCS use for less than a year does not affect attained adult height. On the other hand, prolonged CCS treatment exceeding a year in duration may lead to irreversible growth impairment [61]. Among boys in particular, CCS dose and age of CCS initiation are associated with delayed puberty [48].

With the timely diagnosis of JIA available today, and the use of biologics, the number of children with short stature is expected to fall. In sJIA in particular, biologics targeting the interleukin (IL)-1β or IL-6 pathway are increasingly used [52]. Biologics appear to restore growth retardation by inhibiting inflammation and limiting CCS daily doses [63]. Uettwiller [60] postulated that growth delay is observed in children with JIA long before biologics are initiated. In fact, although biologic treatment normalizes growth velocity, growth delay does not appear to be fully corrected [60]. Furthermore, when more than one biological agents are prescribed, this is associated with poorer growth [59,60]. Subsequently, biologic therapy is often insufficient in restoring normal growth velocity, indicating that rhGH might be required [59].

In a recent proof-of-concept study, Belgian scientists [52] suggested that stature and adipose tissue stores in sJIA can be restored with a hormonal combination therapy including (i) a GnRH analog (triptorelin) to postpone the onset of puberty until a minimum height is attained (or until prepubertal growth spurt is exhausted), and (ii) rhGH at doses of 50 μg/kg/day to promote height gain, once inflammation is controlled and high GCC doses are not required. Of note, rhGH is the cornerstone of growth delay treatment in patients with JIA who have received GCC [64]. Bechtold et al. [50] revealed an increase by 1.5 in the total pubertal growth of rhGH-treated patients in comparison to patients not receiving rhGH, leading to an improved final height. Thus, for the maximization of final height, rhGH treatment must be initiated early on, in order to reduce the height deficit observed at the onset of puberty.

David and associates [51] followed patients with JIA on rhGH treatment to adulthood and showed that median adult height still remained below target height. The authors concluded that long-term rhGH treatment increases growth spurts in JIA; however, it cannot fully restore the genetic growth potential of these children [51]. When the growth velocity of children on rhGH therapy was recorded [29] rhGH was efficient in increasing IGF-1 and IGFBP3 plasma concentrations to values above normal thresholds, improving growth velocity. However, one year after the cessation of rhGH therapy, growth velocity fell to pretreatment values and HAZ was lower than prior to the initiation of rhGH treatment. Nonetheless, rhGH appears to reduce the deficit in stature that occurs during the active phase of JIA, producing an adult height that is closer to the genetically determined height [65]. Overall, on rhGH treatment, catch-up growth markedly varies depending on the severity of inflammation and the CCS doses administered during treatment [64]. According to a systematic review [25] most studies evaluating the effect of rhGH on children with JIA reported positive effects on growth; however, great variability in the treatment response is observed. Suffice it to say, the early initiation of rhGH therapy has been shown to be more effective in maintaining normal growth [64], and the use of combined therapies in parallel to rhGH appears promising in further accelerating growth velocity. It is still unclear, however, whether greater rhGH doses at remission, with patients on biologic therapy post-CCS, can confer a prepubertal growth acceleration, comparable to that observed among GH-deficient children [47].

TNF-α antagonist therapy can also ameliorate growth velocity in JIA [64]. However, the use of etanercept (ETN), a recombinant human soluble TNF-α receptor fusion protein, has been shown to have limited efficacy [64]. In the British Society for Paediatric and Adolescent Rheumatology Etanercept Cohort Study [53], 191 children with JIA treated with ETN were followed for two years. All participants exhibited an improvement in HAZ from baseline; however, a lower baseline HAZ and the use of no oral CCS at baseline were associated with improved HAZ at two years [53]. When the effects of tocilizumab (TCZ), an anti-interleulin-6 (IL-6) receptor monoclonal antibody were examined [35] TCZ was efficient in improving HAZ velocity one year post-baseline. Reduction in CCS use was associated with further improvement in the observed HAZ [35].

Collectively, the literature indicates the important problem of correcting growth delay in children with JIA. Furthermore, the heterogeneity in disease manifestations, therapeutic approaches and medication responses make it difficult to suggest safe, horizontal regimes to exploit the genetic growth potential reserve in JIA.

### 3.2. Undernutrition

Apart from faltering growth and short stature, underweight is also prevalent in children with JIA. Knops [42] suggested that children with JIA exhibited lowed fat-free mass (FFM) compared to controls, except from the oligo- and polyarticular patients, who demonstrated FFM values within the normal range. In general, a lower body mass index (BMI) z-score (BMIz) is observed compared to healthy children [37], and this has been associated with greater disease activity [66]. Research indicates that between 8.3–30% of children with JIA suffer from malnutrition [67,68,69], with dietary intake factors, disease subtype, more affected joints with restricted range of motion (ROM) and younger patient age consisting of effectors of lower BMIz [67].

Most studies, however, suggest that not all JIA subtypes are associated with the same malnutrition risk and/or severity. Young patients with polyarticular disease demonstrate more signs of malnutrition compared to the rest of the subtypes [54,70]. Więch and associates [70] assessed malnutrition through phase angle score, a proxy for nutritional status assessment [71], and a reliable prognostic measure of disease [72]. Among children with JIA, phase angle, percentage of body cell mass and muscle mass were lower compared to that of healthy children [70]. When a subgroup analysis was performed, youngsters with polyarthritis exhibited lower phase angle and muscle mass compared to healthy children; however, no significant differences were observed between children with oligoarthritis and controls, indicating that the pathophysiology of polyarthritis is associated with a greater malnutrition risk. In a similar study, lower total body water was observed in more nutritionally deprived patients with JIA, indicating lower lean mass [54].

In a UK-based case-control study [54], 18.1% of children with JIA demonstrated body weight below the third percentile. Mid-upper arm circumference (MUAC)—an proxy for estimating body fat stores—was below the fifth percentile in 36.4% of the sample. Again, children with polyarticular disease showed significantly more signs of malnutrition than patients with pauciarticular disease, including lower stature, body weight, MUAC, body fat (as a % of body weight) and total body water [54].

Undernutrition has a variety of short- and long-term developmental, behavioral and physiologic effects on children, including increased susceptibility to central fat accumulation, reduced fat oxidation and resting energy expenditure (REE) levels, IR, hypertension and dyslipidemia in adulthood, and a reduced capacity for manual work [73,74]. Thus, although specific screening tools for malnutrition in JIA do not exist to date, frequent weighing of children and the use of appetite enhancers to improve nutritional intake are warranted. Furthermore, the available evidence indicates the lack of JIA-specific dietary intervention trials targeting undernutrition, using enhanced protein formulas, high-energy diets, dietary supplements or combination medical nutrition therapy (MNT).

### 3.3. Overweight and Obesity

Apart from undernutrition though, the other end of malnutrition spectrum, namely, overweight and obesity, is also apparent in children with JIA, in particular those with low disease activity [75,76,77] (Table 2). Young patients with JIA are more frequently overweight/obese than healthy controls, exhibiting greater serum leptin concentrations even after adjustment for fat mass [77]. An increased risk for the development of metabolic syndrome (MetS) has also been noted among youngsters with a JIA diagnosis [78]. The pathophysiological link between obesity, adipokines and cytokines released from the adipose tissue and JIA is not yet clearly understood [79].

Obesity and body weight accumulation is greatly dependent on the environment and the genetics. The genetic link of obesity is apparent in a recent study, where 80% of the overweight children and adolescents with RMDs had parents who were also overweight [87]. In Brazil, approximately 1/5 (21%) of the youngsters with JIA were classified as overweight/obese [68]. When on remission children with JIA exhibit higher measures of central and peripheral adiposity, a greater prevalence of being overweight, including obesity (30%), and larger biceps skinfolds compared to their healthy peers [75]. Disease activity or JIA subtype does not appear to affect body composition, energy intake (EI), or other nutritional biomarkers. Despite the fact that disease activity does not appear to be related to adiposity [66,81,83], it still contributes to the degree of functional impairment and the response to treatment [79]. Similar findings were also reported in Brazil [76], with age, disease subtype, number of affected joints, months since diagnosis and use of CCS or MTX not affecting adiposity. Nonetheless, within the sample, girls with JIA exhibited greater BMIz, body fat (% body weight), trunk fat and fat mass index (FMI) compared to healthy controls [76]. Furthermore, patients with JIA demonstrated lower muscle strength and total bone mineral density (BMD) compared to the control group, although no differences were noted regarding cardiorespiratory fitness (CRF) and body composition.

Only one study [84] reported a positive association between adiposity and disease activity. According to the results, overweight/obesity in JIA correlated with the duration of biological therapy and PA levels, while the FMI of patients was associated with age, the 27-joint Juvenile Arthritis Disease Activity score (JADAS-27) and PA levels [84].

It has been suggested that obesity may also negatively influence the course of the disease, as well as the response to treatment [81]. Collectively, these studies suggest that the changes observed in the body composition of children with JIA increase the risk for developing hypertension, DM and CVD [76]. According to Held [79], the development of early atherosclerosis and CVD in children with obesity consists of an important complication. Furthermore, given that children with JIA often demonstrate IR, higher triglycerides (TG) concentrations, greater systolic blood pressure and early atherosclerosis, as evidenced by ultrasonographic evidence [88], a comprehensive treatment approach targeting overweight and obesity is important for improving disease prognosis and reducing future CVD-related complications.

## 4. Low Physical Activity (PA) Levels

Due to the nature of JIA, children with a diagnosis are, in general, less physically active than their healthy peers [89]. With the environment being an important contributor to PA levels, 56% of the less active children with JIA seem to have parents with similar PA levels [87]. In Canada [89], children with JIA reported participating in less daily moderate-to-vigorous-physical activity (MVPA) than healthy controls, with peak oxygen consumption (VO_2 max_) gradually decreasing with ascending age. Furthermore, patients with JIA demonstrate lower muscle strength as an epiphenomenon of the reduced PA levels [90]. According to Rochette [91], lipid oxidation rates are lower in JIA and respiratory exchange ratios beyond 50% of VO_2 max_ are higher compared to healthy peers, both indicative of metabolic disturbance during exercise, even at times when the disease is inactive.

Furthermore, the observed lower levels of PA are associated with increased functional disability, with patients avoiding positions commonly associated with joint inflammation, intra-articular vascularity and pain [92]. As a result, they tend to apply greater initial hip flexion and lower knee and hip extensions during terminal stance [92].

Low levels of PA can contribute to disease and disability by increasing body weight accumulation and the number of comorbidities associated with overweight (DM, hypertension, etc.), catapulting CVD risk [93,94]. In a study using data from the German National Registry [86], low (or lack of) participation in sports was associated with greater chances for overweight and obesity among children with JIA. Furthermore, low PA is associated with sarcopenia, dynamopenia, low CRF and osteoporosis, as well as with a variety of mental health issues, including anxiety and depression [93,94]. As per Bohr [95], the inactive lifestyle observed in young patients with JIA appears to be a pivotal contributor to the development of subclinical atherosclerosis; thus, the promotion of an active lifestyle during childhood and adolescence is an eminent necessity.

## 5. Gastrointestinal Manifestations

Microbial dysbiosis and gut permeability have been shown to contribute to the inflammation and the immunological imbalance in a synergistic manner [96]. With JIA manifestations often being extended at the GI system, common relevant symptoms include eosinophilic infiltrates [97], gut dysbiosis and small intestinal bacterial overgrowth (SIBO) [96], GI inflammation (mostly affecting the colon (80%)) [97] and autoantibodies indicative of inflamed GI mucosa [98]. Patients with JIA frequently report suffering from chronic abdominal pain [97,99], constipation and diarrhea [100], all of which have been associated with MTX and SSZ use [101]. In parallel, sucrose excretion is increased compared to that of healthy controls, suggesting the existence of gastric and intestinal mucosal lesions (leaky gut) [99]. The observed damage of the intestinal lining is further aggravated as the disease progresses [102]. Among the JIA subtypes ERA is associated with more severe and diverse GI symptoms compared to the rest [98].

Fecal calprotectin (fCal) levels, a marker used in inflammatory bowel diseases (IBD) screening, has been reported to exceed the norm levels in approximately 1/3 of children with JIA [100,103], although extensive non-steroidal anti-inflammatory drugs (NSAIDs) has also been suggested to alter gut inflammation and increase fCal levels [104], although not all studies appear to agree on this issue [105]. Among distinct disease subtypes, ERA is the type associated with greater fCal concentrations [105]. Rare cases of patients with sJIA who were subsequently diagnosed with IBD have also been reported [106], mainly among those with a relevant family history.

A variety of in vivo and in vitro studies indicate that ONS with probiotics can exert immunomodulatory effects through multiple pathways, by (i) regulating intestinal immune function and tampering down inflammation, (ii) inhibiting the entrance of pro-inflammatory gut cytokines to the joints, (iii) preventing the progression of intestinal permeability (leaky gut) and the translocation of gut bacteria, and by (iv) inhibiting the production of autoantibodies inside the inflamed gut [107,108]. According to a recent meta-analysis probiotic supplementation improves JIA-related symptoms [107]; however, routine probiotic supplementation is not recommended by the scientific societies.

## 6. Dyslipidemia

Barsalou [109] postulated that in JIA, the enhanced atherogenic profile of patients is promoted by the dual exposure to both traditional and non-traditional CVD risk factors. In this population, the inadequate intake of energy, paired with the physical inactivity, overweight, chronic inflammation, autoantibodies and medication use (GCC in particular), tend to aggravate dyslipidemia [69]. Children on EΤΝ therapy exhibit elevated total cholesterol (TC), low-density lipoprotein cholesterol (LDLc) and high-density lipoprotein cholesterol (HDLc) levels, whereas, on the other hand, TG and atherogenic index (TC/HDLc) are decreased, evolving to a less-atherogenic profile post-anti-TNF-α treatment [110]. Similar improvements were also shown following MTX treatment alone, or in combination with immunobiological agents [111]. Disease duration does not appear to be related to the observed dyslipidemia [112].

In Brazil, 83.3% of the patients with JIA demonstrated dyslipidemia based on the TC, HDLc, LDLc, TG, non-HDLc, apolipoprotein A_1_ (ApoA_1_), and apolipoprotein B (ApoB) concentrations [68]. Among JIA subtypes, a more atherogenic profile is manifested in sJIA [113,114]. The observed dyslipidemia appears to be aggravated in bouts of elevated disease activity [113]. During the active stage of JIA [115], children with the systemic and RF-positive polyarticular subtype present a reduction in HDLc and a concomitant increase in TG levels. These changes in lipid profile show improvement during disease remission. Inevitably however, dyslipidemia is also associated with increased cardiometabolic risk, as indicated by the elevated uric acid (UA) concentrations [116] observed among patients with JIA.

Nonetheless, aside from the observed disturbances regarding HDL particle distribution, non-lipid transporting activities and cholesterol efflux are also altered [117]. As a result, patients with JIA exhibit reduced arylesterase activity and endothelial cell migration compared to healthy controls. In parallel, macrophages exposure to serum from patients with JIA revealed a smaller increase in the mRNA expression of ATP binding cassette A1 and a greater increase in the expression of both ATP binding cassette G-1 and SR-B1, compared with healthy controls [117].

Interestingly, results from the Lehigh Valley Health Network (LVHN) [118] showed that, despite that fact that most scientific societies advocate for the frequent (every three months) lipid screenings of patients with RMDs [119], approximately one third of the patients on JAK and IL-6 inhibitors fulfill the indications for the initiation of lipid lowering therapies, including lifestyle modifications, but are left untreated.

## 7. Bone Health

Systemic autoimmune disorders such as JIA can affect the skeletal system, reducing BMD and increasing the risk of fragility fractures during childhood [120]. In parallel, pharmacotherapy, and in particular CCS use has been shown to affect bone health and BMD deposition. As a result, lower total BMD [90,121,122,123] and increased bone resorption as measured by greater carboxyterminal telopeptide (CTX) of type I collagen levels are observed in patients with JIA [77]. These marked deficits in BMD coincide with reduced bone strength and are associated with disease severity and duration [124]. Greater CCS use, TNF-α blocking agents and sJIA are associated with extended bone deficits [123,125]. On the other hand, rhGH treatment seems to improve bone characteristics, including bone mineral content (BMC) in children with JIA and short stature [126]. Table 3 presents the results of randomized controlled trials (RCTs) delivering dietary interventions in children with JIA, with the aim of improving bone health.

In a Calcium (Ca) ONS RCT, Carrasco et al. [127] randomized children with JIA to Ca and vitamin D supplementation, or vitamin D alone, for a total of two years. The results revealed that, among the arm receiving combined Ca and vitamin D ONS, serum levels of 1,25-dihydroxyvitamin D (25(OH)D), osteocalcin, parathyroid hormone (PTH), and urine Phosphorus (P) were lower, whereas no incidences of hypercalciuria were noted on spot testing based on the urinary Ca-to-creatinine ratio. The authors concluded that ONS with Ca met the physiologic needs of children, causing an increased Ca urine loss. In a similar trial, Lovell et al. [129] showed that 24 months of Ca supplementation significantly improved BMD in children with JIA compared to placebo; however, participant’s sex, initial BMD, Tanner stage, treatment adherence and body composition were factors explaining the higher BMD in the Ca-arm. In a cross-over manner, Hillman [128] suggested that the percentage of true Ca absorption in JIA is, in fact, in the low range and not affected by any treatment with ONS (placebo, vitamin D_3_, Ca, or combination of vitamin D_3_ + Ca). Thus, although vitamin D_3_ at 2000 IU/day may increase serum 25(OH)D and Ca levels, it does not confer any improvements in bone mass accretion. In parallel, supplementation with Ca (1000 mg/day) was similarly unsuccessful in improving bone mass.

Tang [131] evaluated the administration of a high oral cholecalciferol dose (2000 IU/day) for 24 weeks, compared to no intervention in children with JIA. At the end of the trial, no differences were noted in the BMD or the JADAS-27 score between the two arms, indicating a lack of effect of vitamin D in improving bone health. Nonetheless, given that dual energy x-ray absorptiometry (DXA) can only partly explain bone quality, it has been suggested that bone abnormalities in JIA might be better assessed using complementary imaging techniques, including peripheral quantitative computed tomography (pQCT) or quantitative ultrasonography (QUS) [125,133].

The available research shows that improving bone health in JIA through supplementation is not always successful. Although serum levels of specific biomarkers might be improved post-supplementation, the level of improvement regarding BMC and BMD is questionable, as a possible adverse event of medication and the low levels of PA.

## 8. Energy Requirements and Dietary Intake

According to Knops et al. [42], although crude resting energy expenditure (REE) appears to be indifferent when comparing patients with JIA to healthy controls, REE correction for body mass and FFM showed a 18% higher REE/kg of body weight among young patients with sJIA and 8% greater REE/kg of body weight for oligo- and polyarticular patients. As a result of these elevated REE levels, patients often report feeling overwhelmingly and unremittingly fatigued [101,134,135], complaining for having inadequate energy irrespective of the PA levels [136].

In an early study [43], using direct calorimetry, decreased energy expenditure (EE) was recorded in comparison to the “ideal” values. Nonetheless, greater EE was observed among children with long-time disease and longer follow-up. Furthermore, greater values of IL-6 and TNF were associated with reduced EE [43].

### 8.1. Studies Assessing Dietary Intake in Children with JIA

Table 4 details all the studies conducted to date, assessing the dietary intake of pediatric patients with JIA. According to the literature more than half (54%) of the children fail to reach energy intake levels to meet their daily requirements [43]. Furthermore, protein-energy malnutrition (PEM) has been previously identified in 36% of the young patients with JIA, irrespective of the subtype [137].

Of note, low dietary intake in JIA can also be the result of mouth-related problems. Dental problems and temporomandibular joint disease can affect the child’s ability to eat, as may functional difficulties resulting from arthritis affecting the upper limb [138]. Mouth sores are also frequently encountered, especially in children on MTX treatment [24].

**Table 4:** Studies assessing dietary intake in children and adolescents with JIA.

First Author	Sample	Recruitment	Design	Dietary Assessment Method	Results
Amancio [139]	*Cases: n* = 41 patients with JRA *Controls: n* = 23 patients’ brothers	Pediatric Rheumatology Clinic, Universidade Federal de São Paulo/Escola Paulista de Medicina, Brazil	Case-control	Food Register	Cu and Zn intake were below the RDA. Compared to controls, serum Cu levels were higher, indicating that the number of inflamed joints is related to its variation. Zn levels did not differ in relation to control or disease characteristics.
Bacon [140]	*Cases: n* = 34 children with JRA *Controls: n* = 9 healthy controls	Rheumatology center of Chil- dren’s National Medical Center, Washington DC, USA	Case-control	Three-day dietary intake records	In patients with sJIA in particular, EI was suboptimal to a greater degree. The mean intake of vitamins and minerals for each subtype met the RDA, with the exceptions of Zn and vitamin E and Fe.
Caetano [69]	*Cases: n* = 48 pediatric patients with JIA *Controls: n* = 22 with JSLE	Pediatric Rheumatology Clinic, UNIFESPEPM, São Paulo, Brazil	Case-control	24h diet recalls	An excessive intake of energy, protein, and lipids was observed in 12.5, 75, and 31.3% of the sample, respectively. On the other hand, 41.7, 8.3, and 31.3% of the patients reported consuming less energy, protein, and lipids than required. Fe intake was suboptimal in 29.2% of the sample, Ca in 62.5%, Zn and vitamin A in 87.5% and vitamin B_6_ in 64.6%.
Gorczyca [141]	*Cases: n* = 66 children with JIA *Controls: n* = 44 healthy controls	(i) Clinic of Paediatrics Rheumatology, Wroclaw Medical University, (ii) Department of Paediatric Pulmonology and Rheumatology, Medical University of Lublin, and (iii) John Paul II Paediatrics Centre, Poland	Case-control	Seven-day dietary record	No differences were noted in the EI from dietary fats and PUFA between children with JIA and controls. No differences were observed in the nutrient intake between sero-negative poly-JIA and oligo-JIA subtypes, or between those with active and inactive disease.
Grönlund [75]	*Cases: n* = 40 children with JIA *Controls: n* = 40 healthy controls	Department of Paediatrics, Turku University Hospital, Turku, Finland	Case-control	Seven-day food diaries	EI (kcal/day) was higher in patients with JIA. No differences were noted in the anthropometric measures, the energy and nutrient intakes between JIA subtypes, active and inactive disease.
Hari [142]	N = 33 patients with JIA	(i) Department of Rheumatology, El Ayachi University Hospital and (ii) Department of Pediatrics, University Hospital of children, Rabat-Sale, Morocco	Cross-sectional	24h diet recall for seven consecutive days	A positive correlation was observed between LBM and dietary carbohydrate intake.
Haugen [143]	*Cases: n* = 15 patients with JIA *Controls: n* = 17 healthy controls	Oslo Sanitetsforening Rheumatism Hospital, Oslo, Norway	Case-control	Dietary records	Average daily intakes of protein, fat, vitamins and minerals were similar in all groups. EI/kg of body weight was higher in the polyarticular than in the pauciarticular type. The intake of thiamine, niacin, Fe, Ca and Zn was below the RDA in all groups. One patient with polyarticular and two with pauciarticular JIA abstained from milk, whereas all controls drank milk daily.
Lewis [144]	*Cases: n* = 10 teens with active JIA, *n* = 9 teens with JIA on clinical remission *Controls: n* = 20 healthy controls	Pediatric Rheumatology clinic, Texas TX, USA	Case-control	Three-day food records	Pain and carbohydrate intake were correlated among teens with active JIA. After controlling for sex, added sugars and carbohydrate intake predicted pain scores in active JIA.
López [43]	N = 91 children with JCA for at least a year	Unidad de Nutrición Infantil, Hospital Universitario Infantil “La Paz”, Madrid, Spain	Cohort (One year)	NR	In most cases, total EI suited WHO allowances and protein intake was high (15 ± 2% of total EI). Among the children who required an extra intake to reach an acceptable nutritional status, 54% did not suit their needs.
Mortensen [145]	N = 38 children with JCA	Children’s hospital, Camperdown, Australia	Cross-sectional	Seven day weighed food record	Mean EI was below the RDI among the systemic and polyarticular patients. Mean intakes of Ca and Zn were below the RDI in all of the polyarticular group. Thirteen (34%) children were taking self-prescribed vitamin and/or mineral ONS, with vitamin C being the most common.
Pereira [87]	*Cases: n* = 30 children with polyarticular JIA *Controls: n* = 41 children with JSLE and *n* = 20 children with DM and their parents	Department of Pediatrics, UNIFESPEPM, São Paulo, Brazil	Case-control	Three 24h dietary recalls	The average EI of children with JIA was 1721.3 ± 294.7 kcal/day. No difference was observed between the EI of parents and children. Regarding the consumption of protein, both parents and children consumed amounts exceeding the RDA, and a correlation was noted between parental and children’s consumption.
Rodrigues [68]	N = 62 children/adolescents with polyarticular or sJIA	Department of Pediatrics, UNIFESPEPM, São Paulo, Brazil	Cross-sectional	Three 24h food recalls	Median EI was 1933.6 kcal/day. None of the patients reported a carbohydrate intake exceeding RDA, although 4.9% had a protein intake above the RDA and 29% consumed more lipids than required. The median intake of SFA and trans fats was 24.4 g and 4.2 g, respectively. The majority (75.8%) of children exceeded the SFA recommendation and 79% exceeded the RDA for trans fats.

Ca—Calcium; Cu—Copper; DM—dermatomyositis; EI—energy intake; Fe—Iron; JCA—juvenile chronic arthritis; JIA—juvenile idiopathic arthritis; JRA—juvenile rheumatoid arthritis; LBM—lean body mass; NR—not reported; sJIA—systemic JIA; JSLE—juvenile systemic lupus erythematosus; RDA—recommended daily allowance; RDI—recommended dietary intake; SFA—saturated fatty acids; UNIFESPEPM—Universidade Federal de São Paulo—Escola Paulista de Medicina; WHO—World Health Organization; Zn—Zinc.

#### 8.1.1. Energy and Macronutrient Intake

In a sample of Brazilian children with JIA, Caetano et al. [69] observed an excess in the intake of energy, protein, and lipids was by 12.5, 75, and 31.3%, respectively, paired with a low Iron (Fe), Zinc (Zn), and vitamin A dietary intake. On the other hand, 41.7, 8.3, and 31.3% of the patients reported consuming less energy, protein, and lipids, than required. The food groups identified as being under-consumed involved milk and derivatives (78%), meat (32%), vegetables (98%) and fruits (84%). As a result, Fe intake was suboptimal in 29.2% of the sample, Ca in 62.5%, Zn and vitamin A in 87.5% and vitamin B6 in 64.6%. Nearly half (42.1%) of the children consumed a juice with added sugar on a daily basis.

#### 8.1.2. Suboptimal Levels of Immunonutrients

In an early study, Johansson and associates [146] showed that girls with JIA exhibited lower plasma Selenium (Se) levels compared to their healthy peers. Amancio et al. [139] suggested that, in children with JIA, serum Copper (Cu) concentrations are related to the number of inflamed joints, and greater compared to that of controls. Similar findings were also reported by Haugen et al. [143], where the concentrations of Zn were lower among children with polyarticular JIA compared to controls, and Cu levels were elevated. Se and Zn are important cofactors of antioxidant enzymes, regulating a plethora of inflammatory and immune responses and important micronutrients, in rheumatic diseases in particular [17]. Aside from participating in the antioxidant defense, they also regulate the innate and adaptive immune responses [17,147,148]. With regard to Se in particular, we are yet unsure if the decreased Se concentrations consist of the residue of malnutrition associated with chronic disease or are due to other factors [17,149].

#### 8.1.3. The Curious Case of Ferritin

With regard to Fe levels, a plethora of studies report low Fe and hemoglobin (Hgb) levels among children and adolescents with JIA [143,150,151,152,153,154,155], despite the dietary Fe intake. In parallel, sideroblasts are also reduced in number, pointing to the fact that, in these patients, Fe does not appear to be sufficiently transferred to the erythroid series, limiting the ability for it to be used by erythroblasts, resulting in an absolute iron deficiency anemia (IDA) [156]. Subsequently, Fe is mainly stored in the form of ferritin, although not in an accessible form, hampering metabolism in JIA [156]. Serum ferritin is a known inflammatory marker, although we are not yet sure if it participates in the development of inflammation, or it simply reflects an elevated inflammatory profile [157]. It has been suggested that serum ferritin arises from damaged cells, as a proxy of cellular damage [157]. As a result, the existence of macrophage activation syndrome (MAS) skyrockets the ferritin values [158,159]. However, many patients with active disease (mainly sJIA) without a MAS diagnosis often demonstrate extreme serum ferritin levels, indicating that a distinct inflammatory pathway may exist that is unique to this JIA subtype [154,159,160]. In sJIA in particular, extremely high ferritin levels may be exhibited, even exceeding 1000 ng/mL [161,162], and may assist in establishing the diagnosis of sJIA [159]. Nonetheless, recently, the ferritin/erythrocyte sedimentation rate (ESR) ratio has been suggested for the identification of MAS in sJIA [163].

Anti-TNF therapy has been shown to improve Hgb and the mean corpuscular volume of red blood cells (MCV) without the use of Fe ONS, with a parallel improvement in the concentrations of serum Fe and percent transferrin saturation [156].

#### 8.1.4. Fatty Acid Intake and Status

During the active disease phase children with JIA [141] exhibit low levels of arachidonic acid and docosahexaenoic acid (DHA), a finding that suggests the possible role of polyunsaturated fatty acids (PUFA) in the pathogenesis of JIA, while pointing out the possible need for supplementation. On the other hand, fat intake of children with JIA is also suboptimal, with a recent study [68] showing that 75.8% and 79% of the youngsters with JIA, respectively, presented a SFA and trans-fat intake greatly exceeding the recommendations.

### 8.2. Considerations for the Assessment of Energy Requirements in JIA

With the exception of one study [69], it appears that children with JIA consume suboptimal amounts of energy and several micronutrients. This could be due to disease-specific characteristics and medication adverse effects, and/or to the lack of specific energy equations to assess the actual needs of patients with JIA, leading to an overestimation of the energy requirements. Thus, it is likely that the use of stress factors for the estimation of energy requirements might not be warranted for children with JIA.

In sJIA in particular, where, apart from inflammation, fever is presented frequently during active disease phases, the estimation of REE becomes extremely difficult. Active disease status is associated with reduced body weight, indicating a hypercatabolic state [66]. Overall, in children and adolescents with JIA, the energy expenditure is affected by the fat-free mass of patients, the underlying inflammation (if any), the growth requirements of the children, the exercise-induced activity and the thermogenic effect of food. Fluctuations in disease activity and inflammation induce concomitant alterations in the REE, and thus, when REE cannot be measured, different equations must be used for children in remission compared to those with exacerbated JIA.

### 8.3. Dietary Interventions for Children with JIA

In a pilot uncontrolled study, Berntson et al. [164] evaluated the effect of an anti-inflammatory diet in children and adolescents with JIA. After four weeks of intervention, the sensation of pain, morning stiffness and inflammatory proteins concentrations were reduced, while, in parallel, physical function, fecal butyrate levels and arthritis were improved in the majority of participants. Previously, Berntson had tested the effect of exclusive enteral nutrition (EEN) in a patient with polyarticular JIA for two periods of almost seven weeks each, several months apart [165]. EEN had a remarkable anti-inflammatory effect that was sustained for several months after the treatment periods.

The importance of MNT as a supplementary treatment in JIA becomes apparent in a recent study, where one third of young patients with JIA reported trialing some “special diet” throughout the course of the disease, and more than half of the parents perceived symptom improvement post-intervention [166]. Overall, research on specific dietary interventions targeting JIA is limited, indicating the need for the conduction of more studies evaluating the effect of different diets on JIA outcomes.

#### 8.3.1. Interventions Targeting Insufficient Vitamin D Levels

Vitamin D is an important modulator of both the innate and adaptive immune systems [167,168]. Several lines of evidence reveal suboptimal vitamin D status in children with JIA [169,170,171,172,173,174,175,176,177,178,179,180,181,182,183,184,185,186,187,188] (Table 5). Kondratyeva and associates [182] suggested that among patients with JIA, vitamin D status does not appear to be not affected by the presence of vitamin D receptor (*VDR*) genetic variants. Similar findings were also suggested in an Italian cohort [177], where, additionally, both the *TT* genotype and the T allele were more frequently observed among patients. Furthermore, *VDR* polymorphisms have also been shown to affect lipid metabolism in JIA, with the *VDR* FokI *FF* genotype in particular, demonstrating protective effects [189].

Some researchers reported the existence of a negative association between vitamin D status and disease activity, or the number of affected joints [173,174,175,181,183,185,187], whereas others suggest that low levels of vitamin D are independent of disease status and particularities [169,172,176,178,180,182,186,188,191]. Recent global geographical mapping of the available research revealed the existence of a north–south geographical gradient in the association between low vitamin D status and increased arthritis activity [170]. A Canadian study [187] was the only one reporting greater 25(OH)D levels among children with JIA compared to the same-age general population, a finding attributed to the more frequent use of dietary supplements containing vitamin D. In parallel, birth seasonality has also been suggested to influence vitamin D status, with children with JIA and vitamin D deficiency/insufficiency being more often born during the fall and winter months [187].

Nonetheless, the observed low levels may partly explain why children with JIA often fail to correct bone-related parameters, despite the use of more effective current drugs [184]. Low levels of vitamin D have also been associated with increased CVD risk, as evidenced by greater carotid intima media thickness (cIMT) and lower flow mediated dilatation of the brachial artery (FMD) of children with JIA [190]. Furthermore, a positive correlation has been shown to exist between 25(OH)D levels and FMD, and an inverse one between 25(OH)D concentrations and cIMT. This may explain, at least in part, why despite more effective current drugs, young patients with JIA do not achieve a bone-normal condition over time. Patients with a more severe form of the disease might require higher supplementation of vitamin D to maintain normal 25(OH)D serum levels.

MTX therapy has also been associated with vitamin D insufficiency in JIA [192] and other chronic diseases [193]; however, no specific biologic explanation has been suggested for this phenomenon. It is possible that suboptimal vitamin D levels are participating in the onset and progression of JIA, but it is also highly likely that children with chronic diseases in general are less active outdoors, limiting vitamin D synthesis. Nonetheless, vitamin D ONS has been suggested as an adjuvant in JIA therapy, aiming to correct for the observed low concentrations and the increased CVD risk associated with JIA [167,168,183,184,194]. Supplementation with higher doses of vitamin D (2000 IU/day) is also recommended as an effective generic adjuvant treatment in active JIA [131]. According to Kondratyeva and associates [182], the prophylactic ONS doses of 500–1000 IU/day and 1500–2000 IU/day do not seem to meet the augmented requirements of children with JIA, as the majority seem to attain low levels, despite ONS use, calling for an update of the evidence behind the ONS recommendations.

#### 8.3.2. Interventions Delivering Supplementation/Dietary Intake with Fatty Acids

Active and short-lasting JIA is associated with lower levels of selected fatty acids, including arachidonic acid (AA) and docosahexaenoic acid (DHA). Furthermore, serum α-linolenic acid (ALA) levels are more elevated in poly-JIA than in oligo-JIA. What is more interesting, though, is that serum n-6 and n-3 levels appear to be negatively related to the active joint count, ESR and CRP concentrations, and positively correlated to the platelet count [141].

In patients with rheumatoid arthritis, meta-analyses suggest that the use of n-3 fatty acid supplements may confer clinical benefits [195]. In the case of JIA, uncontrolled clinical trials indicate that ONS with n-3 FA can reduce the daily requirements of NSAIDs, improve the clinical manifestations associated with the disease and lower the degree of inflammation [196,197,198] (Table 6). Furthermore, ONS with n-3 PUFA also induces improvements in several immune parameters, in particular Immunoglobulin A (IgA), Immunoglobulin M (IgM), CD8, CD4/CD8, CD22, IL-1, and IL-4 concentrations, stabilizes lymphocyte cell membranes and activates the organoid level of intracellular regeneration, as indicated by the circulating immune complexes (CIC) [198]. The only RCT performed on pediatric patients with JIA randomized them either to ibuprofen treatment alone, or in combination to a diet with a high omega-3 PUFA content [199]. After five months of intervention, the ibuprofen doses were reduced in the arm receiving ibuprofen in parallel to the high-n-3 diet, although the result was not different compared to that of controls [199].

## 9. Conclusions

**Figure 1:**
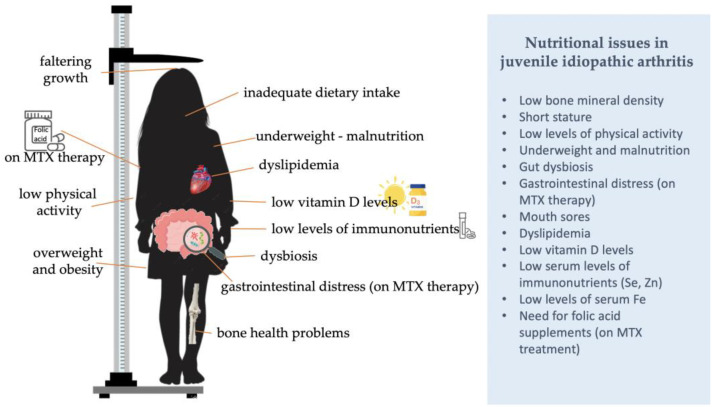
Common nutritional issues in children and adolescents with JIA.

Due to the age of onset, the complexity of the disease and the associated medication, children with JIA are prone to the development of several nutritional issues, warranting expert monitoring. Dietary factors are important regulators of the physiological development of children and adolescents, and in those with a JIA diagnosis their impact on growth, body weight status, bone health, inflammation, and disease activity is augmented (Figure 1). In parallel, most studies indicate a suboptimal dietary intake among children with JIA, and the existence of several micronutrient deficiencies, all of which could be corrected with the appropriate dietary education conducted by experts. For several nutrients, it appears that the recommended allowances fail to meet the requirements of children with JIA. Thus, a multi-disciplinary effort including dietitians and rheumatologists should re-evaluate these recommendations and set JIA-specific targets with regards to the energy, protein and vitamin D intake of young patients in particular.

Collectively, it appears that although MNT can aid in improving JIA prognosis, the number of studies delivering dietary interventions remains low. More recently, the importance of nutrition in JIA has been acknowledged, with a couple of reviews and opinion papers being published [200,201], although the present work consists of the most comprehensive review conducted to date. In parallel, patients and health providers would greatly benefit from the development of specific nutrition and lifestyle guidelines for the management of JIA. The development of a food pyramid, as recently conducted for rheumatoid arthritis [202], but with the use of the Grading of Recommendations Assessment, Development and Evaluation (GRADE) system [203], might be of great use for those involved in the management of JIA.

As the link between nutrition and JIA prognosis is bidirectional and strong, dietitians should be educated on JIA and work closely with rheumatologists and other health professionals in an effort to improve the health of patients in a holistic manner, achieve and sustain maximum growth velocity. Furthermore, rheumatologists would greatly benefit from the inclusion of a specialist dietitian in their team, in an effort to identify nutritional issues in a timely manner and offer the best available MNT in each case. 

## Figures and Tables

**Table 1:** Research on growth disorders in children with JIA.

First Author	Origin	Design	Duration	Participants	Male/ Female Ratio	Results
Bechtold [50]	Germany	Case- control	Until final height	*Cases: n* = 39 children on rhGH and *Controls: n* = 24 not on rhGH	29/35	Greater final height was achieved with rhGH treatment. Final HAZ was determined by age, difference to target stature at puberty onset, and height gain during puberty.
David [51]	France	Prospective cohort	NR	N = 58 patients with JIA (53 on rhGH)	NR	Adult height was available for 48 patients, 8.6 years post-rhGH initiation, however, median adult height was still below target stature. Determinants of growth included age and height at rhGH initiation, and mean CRP levels during follow up.
de Zegher [52]	Belgium	Prospective case series	Until adulthood	N = 2 patients with JIA on biologics	1/1	When rhGH therapy (≈50 μg/kg/day) was added in an on-biological, post-GCC, inactive phase, marked prepubertal growth accelerations were observed, comparable to the “catch-up” responses observed in GH-deficient children.
Kearsley-Fleet [53]	UK	Prospective cohort	2 years	N = 191 ETN-treated patients with JIA (median age 11.0 years)	35/65	Baseline mean HAZ was −0.74 ± 1.4. After 2 years HAZ increased to −0.45 ± 1.4. HAZ improvement was associated with lower baseline HAZ and no use of oral CCS at baseline.
Lofthouse [54]	UK	Case- control	N/A	*Cases*: *n* = 22 children, consecutive patients with JIA (*n* = 7 pauciarticular, *n* = 15 polyarticular) *Controls*: *n* = 22 age- and sex-matched controls	5/17	22.7% were below the third pc for height, with children with polyarthritis being more affected.
Machado [37]	Brazil	Case- control	N/A	*Cases*: *n* = 44 girls with JIA *Controls*: *n* = 59 healthy controls aged between 8 and 18 years	0/44	HAZ was lower in girls with JIA compared to controls. These values differed significantly in Tanner stage II. Three (6.8%) girls with JIA had HAZ <−2SD. Girls with polyarticular JIA and higher cumulative GCC doses were more likely to have short stature.
McErlane [55]	UK	Retrospective cohort	3 years	N = 568 children with JIA (median age 7.4 years)	35/65	Height at 3 years was within the population norm, however, as a cohort, children with JIA showed a growth reduction in height over the first 3 years of diagnosis. Patients with the lowest HAZ at diagnosis were more likely to show improvement at 3 years.
Miyamae [35]	Japan	Prospective cohort	NR	N = 45 patients with sJIA (8.1 ± 4.2 years) on TCZ	17/28	Baseline HAZ was inversely related to disease duration. Improvement in Δ HAZ velocity was seen from 1 year pre- to 1 year post-baseline. Reduction in CCS use was associated with improvement in HAZ velocity.
Moráis [43]	Spain	Prospective cohort	1 year	N = 91 children with JCA (43% pauciarticular, 26% polyarticular, 20% systemic and 11% others)	NR	In 18.7% of the participants, growth rate was retarded, especially in the sJIA and CCS-treated groups. Within the sample, 11% of children had a low Waterloo index.
Polito [56]	Italy	Prospective cohort	4.9 +/- 2.8 years	N = 58 patients with JRA, who had never received CCS	15/43	In systemic and polyarticular JRA a negative association was noted between disease duration and the Δ in HAZ, and between the latter and cumulative periods of active disease. The longer the disease duration, the higher the degree of functional joint involvement. In systemic and polyarticular JRA age of puberty is risk factor for stature impairment.
Saha [46]	Finland	Prospective cohort	4 years	N = 64 prepubertal children with mild-to-moderate JIA	21/43	Preceding the diagnosis, children were slightly taller than their healthy peers, growing at a faster rate. During the 1^st^ year of treatment growth velocity decreased, but at further follow-up, it returned to the pretreatment levels. Growth was aggravated among patients with polyarticular JIA. The cumulative total GCC dose influenced growth.
Simon [36,57]	France	Prospective cohort	NR	N = 24 patients with JIA on steroid therapy	NR	A height loss exceeding 2SD during the first years of the disease was noted, correlated with the duration of PRED therapy. After remission and discontinuation of PRED, 70% achieved catch-up growth, but 30% showed a persistent loss of stature. Mean final height correlated with mean stature post-CCS treatment and was different between those attaining catch-up growth, or not.
Simon [58]	France	Prospective cohort	3 years	N = 13 patients with JIA on rhGH (0.46 mg/kg/week)	NR	Median growth velocity increased from 2.1 to 6.0 cm/year in the 1^st^ year of rhGH therapy and remained above baseline in the 2^nd^ year. HAZ did not change, but growth response varied across patients.
Touati [29]	France	Prospective cohort	2 years	N = 14 patients with severe systemic and/or polyarticular JCA, treated with rhGH (1.4 U/kg/week) for a year	NR	rhGH therapy increased IGF-1 and IGFBP3 levels above normal. All patients showed an increase in growth velocity. In the children who were monitored for a year post-rhGH cessation, growth velocity fell to pretreatment levels, and HAZ at the end of the 2^nd^ year was lower than before treatment.
Uettwiller [59]	France	Retrospective cohort	6 months	N = 100 rhGH-naïve children with JIA (7.1^M^ years (range: 1.6–15.7) at the onset of biologic treatment)	33/77	Patients who required several biologics and those with sJIA had a lower growth velocity post-initiation of biologic treatment. At the last follow-up, 18% of the patients had low growth velocities and 19% were below −2SD, or shorter than genetically programmed.
Uettwiller [60]	France	Prospective cohort	NR	N = 100 prepubescent patients with JIA at onset of biologic treatment (4.26 ± 0.31 years)	NR	More than one biological agent was linked with poor growth. At last follow-up, 10 children had a growth delay and 6 were treated with rhGH.
Wang [61]	Taiwan	Prospective cohort	NR	N = 33 patients with JRA (*n* = 14 with <1 week of systemic CCS therapy, *n* = 13 on CCS therapy for >1 week but never continuously for > 12 months, and *n* = 6 on long-term CCS)	NR	The greatest difference between adult height and target height was observed in those on long-term CCS therapy.
Zak [45]	Denmark	Prospective cohort	18.7 to 46.9 years	N = 65 patients with JIA (32.2 years)	13/52	A HAZ < −2SD was present in 10.7% of patients, all with polyarticular JIA. Polyarticular and sJIA, systemic CCS use and Steinbro-cker functional class II–IV in 1979, were associated with short stature. Disease severity predicted low armspan and final height.

Δ—change; CAPS—Childhood Arthritis Prospective Study; CCS—corticosteroids; CRP—C-reactive protein; ETN—etanercept; GCC, glucocorticoid; GH – growth hormone; HAZ—height-for-age z score; JCA—juvenile chronic arthritis; JIA—juvenile idiopathic arthritis; JRA—juvenile rheumatoid arthritis; IGF-1—insulin-like growth factor-1; IGFBP3—Insulin-like growth factor-binding protein 3; PC- percentile; PRED—prednisone; rhGH—recombinant human growth hormone; SD—standard deviation; sJIA—systemic juvenile idiopathic arthritis; TCZ—tocilizumab.

**Table 2:** Primary research on overweight and obesity among children with JIA.

First Author	Recruitment	Population	Weight Status Classification	Results
Amine [80]	Department of Rheumatology, University Hospital of Rabat-Sale, Morocco	N = 58 patients with JIA	IOTF	Twenty-four patients (41.4%) were overweight and 13 (22.4%) were obese. Overweight/obesity were more prevalent in older patients with more functional impairment and active disease (higher VAS). No relationships were observed between adiposity, JIA subtype or CCS treatment.
Caetano [76]	Paediatric Rheumatology Division (Unifesp)	*Cases*: *n* = 42 girls with JIA *Controls: n* = 35 healthy controls	WHO z-scores	An equal proportion of the girls with JIA were overweight and obese (11.9%). Girls with JIA had higher median BMIz scores.
Giani [81]	Rheumatology Unit, A. Meyer Children’s Hospital, University of Florence, Florence, Italy	N = 110 patients with JIA	CDC	Baseline BMI was ≥5th and ≤84th pc in 80 patients, 85–94th in 27, and ≥95th in 3. No associations were noted between BMI and ESR, CRP, or number of active joints at baseline. Involvement of the joints of lower limbs was greater in patients with overweight/obesity.
Grönlund [75]	Department of Paediatrics, Turku University Hospital, Finland	*Cases*: *n* = 40 children with JIA *Controls: n* = 40 healthy children	NR	Obesity/overweight was more common in JIA (30% vs. 12.5%).
Milatz [82]	Centre of Reference for Paediatric and Adolescent Rheumatology, University Hospital Centre Zagreb, Croatia	*Cases*: *n* = 3334 children-adolescents with JIA *Controls: n* = 3334 sex- and age-matched pairs	German reference system	Overweight and obesity rates were 8.8% (vs. 8.5%) and 6.1% (vs. 5.7%), respectively. Higher rates of overweight were observed in adolescent patients than affected children. Patients with ERA (22%), PsA (21%) and sJIA (20%) had the highest overweight/obesity rates.
Neto [66]	Rheumatic Diseases Portuguese Register	N = 275 patients with JIA	WHO pc	The prevalence of overweight and obesity was 15.3% and 10.5%, respectively.
Pelajo [83]	Department of Pediatric Rheumatology, Floating Hospital for Children, Tufts Medical Center, USA	N = 154 patients with JIA	2000 CDC growth charts	Obesity was found in 18% of the patients, whereas 12% were overweight. There was no association between obesity and JADAS-27, physician’s assessment of disease activity, parent’s assessment of child’s well-being, ESR, active joint count, or CRP.
Rego [84]	Pediatric Rheumatology Department, Hospital Regional Universitario de Málaga, Spain	*Cases*: *n* = 80 children with JIA *Controls: n* = 80 healthy age- and sex-matched volunteers	WHO z-scores	Patients with a high inflammatory activity (JADAS-27 > 4.2 for oligoarticular JIA or >8.5 for polyarticular disease) had higher values of BMI. Overweight/obesity was associated the duration of biological therapy.
Rodrigues [68]	Department of Pediatrics, Universidade Federal de São Paulo, Brasil	N = 62 children with JIA	WHO pc	21% of the patients were classified as overweight/obese.
Sellami [85]	Mongi Slim Hospital, Rheumatology, Tunisia	N = 55 patients with JIA	French curves	Twenty-two patients (40%) were overweight and 15 (27%) were obese. Severe functional limitation, sJIA, and active disease were the parameters most correlated with obesity.
Schenck [86]	National Paediatric Rheumatological Database (Germany)	*n* = 2951 children with JIA (2003), *n* = 3903 (2005) and *n* = 5667 (2012)	Curves for German children	The rate of overweight decreased from 14.2% in 2003 to 8.3% in 2012. sJIA and ERA were more likely associated with overweight. The use of high-dose CCS, lower functional limitations, and a low level of school sports participation were predictors of overweight.

BMI—body mass index; CCS—corticosteroid; CDC—Centers for Disease Control; CRP—C-reactive protein; ERA—Enthesitis-related arthritis; ESR—erythrocyte sedimentation rate; JADAS-27—Juvenile Arthritis Disease Activity Score 27; JIA—juvenile idiopathic arthritis; IOTF—International Obesity Task Force; NR—not reported; pc—percentiles; PsA – psoriatic arthritis; sJIA—systemic JIA; VAS—visual analogue scale; WHO—World Health Organization.

**Table 3:** RCTs delivering dietary interventions for improving bone health in children with JIA.

First Author	Origin	Population	Intervention(s) and Comparator(s)	Intervention Duration	Outcomes	Results
Carrasco [127]	USA	N = 198 patients with JRA	(1) ONS with Ca (1000 mg) + vitamin D (400 IU) daily (2) ONS with placebo + vitamin D (400 IU)	24 months	Serum PTH, Ca, OC, 25(OH)D, urine P levels	Patients with ≤4 affected joints had higher serum levels of Ca and PTH. Ca-treated patients with ≤4 joints with active disease had lower levels of OC. At follow-up, levels of 1,25(OH)2D, OC, PTH, and urine P were lower in the Ca-receiving arm.
Hillman [128]	USA	N = 18 children with JRA	(1) placebo (2) ONS with vitamin D_3_ (2000 IU/day) (3) ONS with Ca (1000 mg/day) (4) ONS with vitamin D_3_ (2000 IU/day) + Ca (1000 mg/day)	6 months each arm	BMC, bone turnover, PTH, 25(OH)D, 1,25(OH)2D levels	Percent true Ca absorption was in the lower-normal range and did change by treatment. ONS with vitamin D_3_ and vitamin D_3_ + Ca increased 25(OH)D levels. Serum Ca levels were improved only in the vitamin D_3_ and vitamin D_3_ + Ca arm. BMC or bone turnover markers did not differ.
Lovell [129]	USA	N = 198 children with JRA	(1) ONS with Ca (1000 mg) + vitamin D (400 IU) daily (2) ONS with placebo + vitamin D (400 IU)	24 months	BMD	At 24 months, BMD was improved in the Ca-receiving arm compared with the placebo. Baseline BMD, sex, Tanner stage, treatment adherence and body composition were factors explaining greater BMD in the Ca-arm compared with the placebo.
Stark [130]	USA	N = 49 children with JRA	(1) usual care (three sessions) (2) six session BI	6 months	Ca intake, total BMC, arms and legs BMC, lumbar spine BMD (DXA)	BI maintained an average Ca intake of 1500 mg/d at 6 and 12 months. This was greater than the baseline level, but not greater than the intake maintained by usual care. The BI induced a 4% and 2.9% greater gain in total BMC than usual care at 6 and 12 months, and a 7.1% and 5.3% greater gain in arms and legs BMC.
Tang [131]	China	N = 36 treatment-naive patients with JIA (pp)	(1) ONS with vitamin D_3_ (2000 IU/day) (2) no ONS	24 weeks	JADAS-27BMDz serum levels of 25(OH)D	No differences were noted between the two arms regarding the change in BMD or JADAS-27 score.
Warady [132]	USA	N = 10 children with RMD and OP (*n* = 6 with JRA), on CCS	(1) Ca ONS (2) vitamin D ONS	6 months	OC, BMD	Spinal BMD improved with supplementation but OC levels remained low throughout the study.

Abbreviations: 1,25(OH)2D—1,25-dihydroxyvitamin D; 25(OH)D—25-hydroxyvitamin D; BI—behavioral intervention; BMC, bone mineral content; BMD—bone mineral density; BMDz—bone mineral density z-score; Ca—Calcium; CCS—corticosteroids; DXA—dual-energy X-ray absorptiometry; JADAS-27—27-joint Juvenile Arthritis Disease Activity Score; JIA—Juvenile idiopathic arthritis; JRA—Juvenile rheumatoid arthritis; IU—international units; P—Phosphorus; PTH—parathyroid hormone; pp—per protocol analysis; OC—osteocalcin; ONS—oral nutrient supplementation; OP – osteoporosis; RCT—randomized controlled trial; RMD – rheumatic musculoskeletal diseases.

**Table 5:** Primary research on vitamin D status in children with JIA.

First Author	Recruitment	Population	Results
Abu-Zaid [190]	Rheumatology and Rehabilitation, Faculty of Medicine Tanta University, Egypt	*Cases: n* = 30 patients with JIA *Controls: n* = 30 healthy age and sex-matched volunteers	Only 23.3% of the patients had adequate vitamin D levels. Vitamin D insufficiency and deficiency were observed in 30% and 46.7%, of the patients, respectively. JIA patients had lower levels as compared to controls.
Bouaddi [173]	Department of Rheumatology, El Ayachi Hospital, Morocco	N = 40 children with JIA	Hypovitaminosis D was observed in 75% of the sample. Levels of 25(OH)D were negatively related to the DAS28 for polyarticular and oligoarticular JIA.
Cetrelli [188]	From three geographically spread regions in Norway	N = 223 participants with JIA	Mean serum vitamin D concentrations were 61.4 nmol/L and 29.6% had insufficient levels. Vitamin D levels differed between regions, age groups, BMI tiers, and seasons for blood sampling. Insufficient vitamin D levels were associated with dentin caries and gingival bleeding. No associations were observed with active or more severe disease.
Çomak [185]	Division of Pediatric Nephrology and Rheumatology, Department of Pediatrics, Akdeniz University, Faculty of Medicine, Turkey	N = 47 patients with JIA	Vitamin D insufficiency and deficiency were observed in 19.1% and 53.2% of the sample, respectively. Overall, the levels of 25(OH)D were <20 ng/mL in 72.3% of the children. There was a negative correlation between vitamin D concentrations and disease activity.
Dağdeviren-Çakır [176]	Division of Pediatric Endocrinology, İstanbul University Cerrahpasa Faculty of Medicine, Turkey	*Cases: n* = 64 patients with JIA, *n* = 36 patients with FMF *Controls: n* = 100 healthy children	There was no significant difference between vitamin D levels during activation and remission periods in the patients with JIA. No significant relationship was found between disease activity and serum vitamin D levels. The levels of vitamin D in JIA and FMF were lower compared to those of healthy children.
de Sousa Studart [172]	Fortaleza, Brazil	*Cases: n* = 50 patients with JIA *Controls: n* = 20 age- and sex-matched controls	Levels of 25(OH)D were similar regardless of JIA type, disease activity/severity (JADAS-27, CHAQ, or presence of joint deformities). Most (52%) patients had optimal vitamin D levels, 40% had sufficient and 8% were deficient in vitamin D. Ethnicity, BMI, season and CCS use did not influence 25(OH)D levels.
Finch [187]	Alberta Children’s Hospital, Cumming School of Medicine, University of Calgary, Canada	*Cases: n* = 164 patients with JIA *Controls: n* = 4027 same-age general population data	Mean 25(OH)D concentrations were higher in JIA than controls, as the first reported more frequent use of vitamin D containing supplements (50% vs. 7%). The prevalence of 25(OH)D deficiency was 6% in both groups. Children with JIA and 25(OH)D deficiency/insufficiency had higher CRP levels and were more often born in the fall/winter.
Kondratyeva [182]	Multicenter in Russia	*Cases: n* = 150 patients with various JIA variants *Controls: n* = 277 healthy children	A high prevalence of low vitamin D was observed among children with JIA (66%) and dietary intake was below expected norms. Prophylactic vitamin D doses of 500–1000 IU/day and 1500–2000 IU/day do not seem to meet the needs of children with JIA. Vitamin D status among children with JIA was not affected by clinical therapies or the presence of *VDR* genetic variants.
Marini [177]	Anna Meyer Children’s University Hospital, Italy	N = 103 patients with JIA	The majority of patients (84.5%) had suboptimal levels, in many cases (84.1%) not solved by ONS. Vitamin D status was independent of *VDR* genotypes. ApaI genotypes were more prevalent in JIA with both the *TT* genotype and the T allele more frequent.
Munekata [191]	Unidade de Reumatologia Pediátrica, Departamento de Pediatria, Brasil	*Cases: n* = 30 patients with polyarticular JIA *Controls: n* = 30 healthy age and gender-matched individuals	No differences were observed in the 25(OH)D, PTH or serum P levels between JIA and control arms. These values were not associated with disease activity, use of medications or BMD. A high prevalence of 25(OH)D insufficiency/deficiency was observed.
Nandi [181]	Department of Pediatrics, Nilratan Sircar Medical College and Hospital, India	N = 40 patients with JIA	A negative correlation was observed between the JADAS-27 score and serum vitamin D levels.
Pelajo [186]	Division of Pediatric Rheumatology, Floating Hospital for Children, Tufts Medical Center, USA	N = 154 patients with JIA	Levels of 25(OH)D were not associated with JADAS-27, or its individual components. Age, ethnicity, BMI and season were related to serum 25(OH)D levels.
Rosiles [179]	Servicio de Reumatología, Instituto Nacional de Rehabilitación, México	*Cases: n* = 37 patients with JIA *Controls: n* = 37 patients with SLE, *n* = 79 healthy controls	Approximately 35.1% of patients with JIA and 31.6% of healthy controls had deficient levels of vitamin D.
Sengler [174]	German Rheumatism Research Center, Leibniz Institute, Germany	N = 360 patients with JIA	Nearly half of the patients had a deficient 25(OH)D status (< 20 ng/mL) in the first sample and ¼ had a deficient level in both samples. Disease activity and the risk of developing JIA-associated uveitis were inversely related to the concentrations of 25(OH)D.
Shevchenko [183]	Department of Cardio-Rheumatology, Institute of Children and Adolescents Health Care, Ukraine	*Cases: n* = 69 patients with JIA *Controls: n* = 15 healthy children	Although reduced vitamin D status was observed in both groups, healthy children demonstrated higher 25(OH)D levels. A significant relationship was observed between the number of active joints and the age of patients, duration of disease, the level of vitamin D in serum, the number of injured joints, and disease activity (JADAS-27).
Stagi [184]	Department of BioMedicine, Section of Rheumatology, Transition Clinic, University of Florence, Italy	*Cases: n* = 152 patients with JIA *Controls: n* = 152 age and sex-matched controls	Patients with JIA exhibit lower 25(OH)D and higher PTH levels.
Stawicki [169]	Department of Pediatrics, Rheumatology, Immunology and Metabolic Bone Diseases, Poland	N = 189 patients with JIA	Vitamin D deficiency was observed in 67.2% of the sample and was independent of sex, disease manifestation, CRP, ESR, ALP, or PO_4_ concentrations. Greater MTX doses corresponded to lower 25(OH)D levels.
Sumi [175]	Pediatric Rheumatology clinic of BSMMU, Bangladesh	*Cases: n* = 30 newly diagnosed cases of JIA *Controls: n* = 30 age and sex-matched controls	Among JIA patients 60%, and among controls 33% showed hypovitaminosis D. Serum 25(OH)D levels were lower in JIA compared to controls and the result was significant in cases of poly-articular JIA and sJIA. Level of serum 25(OH)D significantly decreased as disease duration continue increased.
Szymańska-Kałuża [180]	Department of Pediatric Cardiology and Rheumatology, Medical University of Lodz, Poland	*Cases: n* = 50 children with JIA *Controls: n* = 28 healthy controls	The concentration of serum 1,25(OH)2D in JIA was lower compared to that of children in the control arm.
Wang [178]	Department of Rheumatology Immunology and Allergy, Children’s Hospital, Zhejiang University, China	*Cases: n* = 53 children confirmed as having JIA *Controls: n* = 106 healthy children	Compared to controls, children with JIA had lower serum 25(OH)D3 levels. The percentage of subjects with severe deficiency in the JIA group was higher than that of controls (17.0% vs. 6.6%). Serum 25(OH)D3 levels were not related to JIA subtypes, ACR Pediatric 30 Score, CRP or ESR.

Abbreviations: 25(OH)D—25-hydroxyvitamin D; ACR—American College of Rheumatology; ALP—alkaline phosphatase; BMD—bone mineral density; BMI—body mass index; BSMMU—Bangabandhu Sheikh Mujib Medical University; CCS—corticosteroids; CRP—C-reactive protein; DAS28—disease activity score 28; ESR—Erythrocyte sedimentation rate; FMF—familial Mediterranean fever; JADAS-27—27-joint Juvenile Arthritis Disease Activity Score; JIA—Juvenile idiopathic arthritis; MTX—methotrexate; P—Phosphorus; PO_4_—phosphate; PTH—parathyroid hormone; sJIA—systemic JIA; SLE—systemic lupus erythematosus; VDR—vitamin D receptor.

**Table 6:** Clinical trials delivering dietary interventions for improving bone health in children with JIA.

**First Author**	**Origin**	**Population**	**Intervention(s) and Comparator(s)**	**Intervention Duration**	**Outcomes**	**Results**
Gheita [196]	Egypt	N = 27 patients with JIA	ONS with n-3 FA (2 g/day)	12 weeks	JADAS-27, ACR response criteria, CHAQ, IL-1, TNF-α, NSAIDs use	An improvement was noted in active joint count, number of swollen joints, JADAS-27, CHAQ, TNF-α, and IL-1 levels. The pediatric ACR response criteria improved in 92.6% of the sample. The daily need for NSAIDs was decreased.
Gollini [197]	USA	N = 16 youngsters with JIA with unremitting pain and inflammation	ONS with Kre-Celazine (alkali-buffered creatine monohydrate and cetylated FA blend)	30 days	Inflammation markers, ROM, pain, CRP and ESR levels	Treatment with Kre-Celazine improved and, in some cases, resolved symptoms among youngsters who had not achieved satisfactory relief from the use of NSAIDs.
Vargová [199]	Slovakia	N = 23 children with JCA	(1) ibuprofen treatment (2) ibuprofen + diet with an increased n-3 PUFA content	5 months	Ibuprofen dose reduction	In the ibuprofen + diet arm, in the course of treatment the original ibuprofen dose declined by 17.3%, while in the control arm there was a decline equal to 6.5%.
Yarema [198]	Ukraine	*Cases: n* = 53 children JRA *Controls: n* = 15 healthy controls	(1) Usual therapy (2) Usual therapy + ONS with n-3 PUFA	24 weeks	CD3, CD4, CD8, CD16, CD22, IgA, IgM, IgG, IL-1, IL-4, IL-6, CIC	A greater improvement of immune parameters was demonstrated in the n-3 PUFA receiving arm. Between the 2 treatment arms improvements were noted in IgA, IgM, CD8, CD22, IL-1, and IL-4 levels.

ACR—American College of Rheumatology; CHAQ—Childhood Health Assessment Questionnaire; CIC—circulating immune complexes; CRP—C-reactive protein; ESR—erythrocyte sedimentation rate; FA—fatty acids; JADAS-27—27-joint Juvenile Arthritis Disease Activity Score; JCA—juvenile chronic arthritis; JIA—Juvenile idiopathic arthritis; IgA—Immunoglobulin A; IgM—Immunoglobulin M; IgG—Immunoglobulin G; IL-1—interleukin 1; IL-4—interleukin 4; IL-6—interleukin 6; NSAID—non-steroidal anti-inflammatory drugs; ONS—oral nutrient supplementation; PUFA—poly-unsaturated fatty acids; ROM—range of motion; TNF-α—tumor necrosis factor α.

## Data Availability

Not applicable.
